# Current advances of *Pichia pastoris* as cell factories for production of recombinant proteins

**DOI:** 10.3389/fmicb.2022.1059777

**Published:** 2022-11-24

**Authors:** Yingjie Pan, Jiao Yang, Jianping Wu, Lirong Yang, Hao Fang

**Affiliations:** ^1^ZJU-Hangzhou Global Scientific and Technological Innovation Center, Zhejiang University, Hangzhou, Zhejiang, China; ^2^College of Chemical and Biological Engineering, Zhejiang University, Hangzhou, Zhejiang, China; ^3^College of Life Sciences, Northwest A&F University, Xianyang, Shaanxi, China

**Keywords:** *Pichia pastoris*, recombinant protein, CRISPR/Cas9, metabolic engineering, modeling

## Abstract

*Pichia pastoris* (syn. *Komagataella* spp.) has attracted extensive attention as an efficient platform for recombinant protein (RP) production. For obtaining a higher protein titer, many researchers have put lots of effort into different areas and made some progress. Here, we summarized the most recent advances of the last 5 years to get a better understanding of its future direction of development. The appearance of innovative genetic tools and methodologies like the CRISPR/Cas9 gene-editing system eases the manipulation of gene expression systems and greatly improves the efficiency of exploring gene functions. The integration of novel pathways in microorganisms has raised more ideas of metabolic engineering for enhancing RP production. In addition, some new opportunities for the manufacture of proteins have been created by the application of novel mathematical models coupled with high-throughput screening to have a better overview of bottlenecks in the biosynthetic process.

## Introduction

During the last three decades, *Pichia pastoris (Komagataella phaffii*) was a common cell factory producing recombinant proteins (RP), which shows larger physiological advantages compared with other commonly used host cells (Zhu et al., 2019; Karbalaei et al., 2020). *P. pastoris* is a methylotrophic yeast (Zahrl et al., 2017), usually grown in dynamic culture systems, in which the cell culture environment presents a constant change and a lot of variable factors affect the productivity of products. It has some specific physiological properties, namely, the ability to grow rapidly at high cell densities (>150 g dry cell weight/liter) in simple media and secrete proteins at high yields under bioreactor conditions (Jahic et al., 2007; Parashar and Satyanarayana, 2016). In general, the advantages of protein production using *P. pastoris* system include higher folding efficiency, high cell density fermentation, strong expression system, genetic stability, and a mature secretion system of secreting proteins to the external environment (by Kex2 as signal peptidase) (Schwarzhans et al., 2017; Karbalaei et al., 2020). The phase of protein initiation was separated with the generation of a large number of cells (Tolner et al., 2006). Actually, *P. pastoris* enjoyed a more frequent application for the heterologous protein production relative to *Saccharomyces cerevisiae* according to one literature survey (Bill, 2014). Driven by the AOX1 promoter, the titer of RP production by *P. pastoris* is higher than 10 g/L, equivalent to 30% of total cell proteins (Duan et al., 2018). Despite the operation easiness and a well-determined process of the *P. pastoris* expression system, it is necessary to optimize the process for achieving the largest RP production.

Historically, *P. pastoris* was first isolated from the exudates of a chestnut tree in France and Guilliermond named it as *Zygosaccharomyces pastoris* (Zahrl et al., 2017). Then, it was classified by Yamada et al. to a novel genus, *Komagataellaor Pichia* (Naumov et al., 2018). More than 40 years ago, It was commercially applied for single cell protein (SCP) production by virtue of the high cell density fermentation process as animal feed additive with the utilization of methanol (Cregg et al., 1985; Cereghino et al., 2002). In the 1980s, *P. pastoris* was first developed as a heterologous protein expression system by using powerful and well-regulated AOX1 promoter (Cregg et al., 1985). One of the first large-scale industrial production processes was established in the 1990s, capable of producing enzyme hydroxynitrile lyase by >20 g of RP/liter of culture volume (Hasslacher et al., 1997). Unlike the Y11430 strain (wild type), GS115 is a common *P. pastoris* strain for protein expression, particularly in industry and medicine (Julien, 2006), which has 2 encoding genes of alcohol oxidase (AOX) enzymes, namely, AOX1 and AOX2. This yeast initially attracted attention because of its native character of utilizing methanol as the sole carbon source, which can be achieved by certain metabolic pathways that involved many special enzymes (Gellissen, 2000). By now, over 5,000 proteins have achieved successful cloning and expression with *P. pastoris* system (Liu et al., 2022). Nevertheless, *P. pastoris* cannot produce or secrete all proteins to such high titers. Under normal conditions, the protein production is obviously lower, especially with the expression of complex proteins which are hetero-oligomers, attached to membrane or easily suffer proteolytic degradation (Ahmad et al., 2014).

Since it was first discovered, various approaches have been applied to reshape *P. pastoris* based on their needs. Although *P. pastoris* has served as a standard platform for production of RPs for many decades, some limitations still exist that prevent it from being a trustable “cell factory” with high productivity and adaptability. Few powerful transcription tools are currently available in *P. pastoris* (Yang and Zhang, 2018), meanwhile, common used promoters of *P. pastoris* such as P_AOX1_ respond to methanol, which has the drawbacks of toxic, flammable, and explosive (Gasser et al., 2015; Vogl et al., 2016). The narrow regulation mode of methanol-inducible promoters severely limits its application scope. Efforts also have been devoted to rewiring this expression host and developing new genetic tools. The beginning to use of random mutagenesis and screening procedures (Liu et al., 1992), is followed by the establishment of selectable markers (Cereghino et al., 2001) and more complex genetic engineering tools such as the Cre-lox recombinase system (Li D. et al., 2017) CRISPR/Cas9 (Gao et al., 2022a) were established over the years. The study summarized the recent advances regarding system and synthetic biology which includes metabolic engineering in the understanding of the phenotypic characteristics exhibited by recombinant *Pichia* and genome editing techniques like the CRISPR-Cas system. Mathematical modeling and application of high-throughput screening were also introduced here for better control of the biosynthesis pathway.

## Host strain engineering

### Improvement of homologous recombination efficiency

Although DNA repair mechanisms in *S*. *cerevisiae* have been deeply understood, there are still many challenges in the exploration of the alternative yeast *P. pastoris* (Ahmad et al., 2014). Compared to *S. cerevisiae*, knock-out cassettes or knock-in expression fragment present a more strenuous and low-efficient targeted integration with homologous arms in *P. pastoris* (Fischer and Glieder, 2019). One reason is that *P. pastoris* uses non-homologous end-joining (NHEJ) as a main DNA repair mechanism when DNA double strand broke (Naatsaari et al., 2012). DNA repair by homologous recombination (HR) offers several advantages over NHEJ, including more efficient screening for positive clones and improved genomic stability (Dalvie et al., 2022). However, *P. pastoris* presents a low HR rate, and a majority of the introduced DNA cassettes are integrated at random sites in the genome by virtue of NHEJ (Wang et al., 2016). Hence, *P. pastoris* gives more favor to the NHEJ pathway than recombination events (HR) for repairing double-strand breaks (DSB) (Fischer and Glieder, 2019). Currently, this issue was addressed by a double knockout of the homogeneous genes *dnl4* (coding for DNA ligase IV) of *Ku 70*, overall increased efficiency to a comparable level of other eukaryotic cells (Ito et al., 2018). Relative to *S. cerevisiae*, in which short overhangs are capable of targeting strongly specific integrations, in *P. pastoris*, long overhangs present a larger efficiency and foreign DNAs may undergo ectopic integration (Vogl et al., 2018a). In order to overcome this issue, HR machinery with multiplex genome integration was introduced from *S. cerevisiae* to *P. pastoris* strains. Core genes related to HR were overexpressed under strong constitutive promoters (i.e., PGAP and PTEF1) and inserted into the *P. pastoris* chromosome. Thus, HR efficiency was enhanced to realize effective one-, two-, and three-loci genome integration as high as 100, ∼98, and ∼81% using ∼40 bp homology arms (Gao et al., 2022b).

The appearance of novel genome editing tools eased the gene disruption and specifically improved the HR efficiency. One good example is CRISPR/Cas9. CRISPR/Cas9 is a powerful genetic editing tool, and has been developed into a mature laboratory technique capable of disrupting certain genes with high efficiency in prokaryotic and eukaryotic systems (Sander and Joung, 2014). It utilizes a nuclease, introducing a DNA double-strand break at the regions complementary to the gRNA sequence under the guidance of a short RNA (guide RNA: gRNA or single guide RNA: sgRNA). Recruiting the cellular repair mechanism helps to seal these breaks, which allows to introduce difference genome modifications, i.e., NHEJ in the case of no template and homology directed repair (HDR) with appropriate repair template (Jiang and Doudna, 2017). CRISPR/Cas9 can be reprogrammed, and different loci can be targeted by changing 20 bp of gRNA (Fischer and Glieder, 2019). In spite of its convenience, CRISPR/Cas9 was first adapted to *P. pastoris* in 2016 (Weninger et al., 2016). Expression of Cas9 usually relies on the use of regular genomic vectors, while nuclear localization as well as 5′–3′ trimming is needed for the single guide RNAs. The identification of this RNA polymerase-III (PolIII) mediated transcription in *P. pastoris* can be easily achieved by small RNA sequences, highly reducing the difficulty of multiplex cloning and expression (Dalvie et al., 2020). Later research further explored the potential of this technique (Weninger et al., 2018; Yang et al., 2020). The deletion of the *Ku70* involved in NHEJ would remarkably improve the HR repair, achieving nearly 100% efficiency for site-directed of gene insertion (Weninger et al., 2018; Liu et al., 2019), but with the help of CRISPR/Cas9 system, HR efficiency could also be 100% for Mxr1 (methanol expression regulator 1) using the same sgRNA without deleting *Ku70* due to the help of stable Cas9 expression through integrative expression (Yang et al., 2020). Furthermore, the HR efficiency was improved by even over 54 times in NHEJ deficient strain by the CRISPR-Cas9 system, which assisted in the incorporation of donor DNA to express heterologous gene (Cai et al., 2021). However, hard to knock out genes such as *OCH1*, where CRISPR/Cas9 showed only 50% mutation efficiency (Weninger et al., 2016). Normal approaches solve this problem such as increasing HR efficiency by addition of hydroxyurea during transformation to stop cell division at the S/G2 phase where the status of HR is rather more active than NHEJ (Lee et al., 2018). Compared to the *Ku70* mutation strain, this method does not reduce transformation efficiency and it is more suitable for indel mutation (Ahmad et al., 2019; Gassler et al., 2019). Recently, another study revealed that *Ku70* mutation negatively impacted the chromosome terminal stability that caused loss of colonies, but over-expression of *RAD52* can drastically improve the efficiency of HR (Cai et al., 2021).

Besides the gene replacement and the targeted insertion, CRISPR/Cas9 can serve for creating indel mutations at targeted genomic loci by virtue of the NHEJ DNA repair mechanism under the condition of no homology target (donor DNA). HDR mediated by CRISPR/Cas9 allows DNA fragments to be effectively integrated into the genome, which is marker-free in selection, and is likely to replace, destroy or label a certain genomic locus (Singh et al., 2017). Recently, one synthetic biology toolkit based on CRISRP was established to ensure that multigene biosynthetic pathways could be integrated as well as assembled into the chromosome of *P. pastoris* with the selection of integration sites for the efficient and stable genome integration. Also, it characterized a panel of constitutive, methanol-inducible promoters, enabling the integration of one locus, two loci, and three loci (efficiency: ∼100, ∼93, and ∼75%, respectively) (Gao et al., 2022a). The most recent applications for CRISPR/Cas9 were shown below in Table 1.

**TABLE 1:** Genetic engineering tools for *Pichia pastoris* through HR,^1^ adapted from Cai et al. (2021).

Strain engineering	Cas 9 expression	sgRNA expression	Genome cutting efficiency	Seamless deletion	Genome integration	Marker needed	Minimal HA^2^ length	References
/	P_HTA1_, episomal	P_HTB1_, episomal	43–95%	2.4%	Single gene (24%)	Yes	1000 bp	Weninger et al., 2016
kuΔ70 for blocking NHEJ	P_HTA1_, episomal	P_HTB1_, episomal	94%	gut1Δ (100%)	/	No	1000 bp	Weninger et al., 2018
kuΔ70 for blocking NHEJ	P_ENO1_, episomal	P_tRNA3_, episomal	93%	/	Three genes (20%)	No	500 bp	Dalvie et al., 2020
kuΔ70 for blocking NHEJ	P_HTA1_, episomal	P_FLD1_, P_AOX1_ or P_GAP_, episomal	75–98%	/	Two genes (58–70%)	No	1000 bp	Liu et al., 2019
					Three genes (13–32%)			
Overexpressing RAD52 and/or deleting MPH1	P_HTA1_, episomal	P_HTB1_, episomal	93%	gut1Δ (90%)	Single gene (43–70%)	No	50 bp	Cai et al., 2021
				faa1Δ (88%)	Three genes at one site (67.5%)			
				faa2Δ (88%)	Three genes at three site (25%)			
/	hCas9, Intergrated	P_HZP–gRNA_, episomal	/	/	Single loci (100%)	No	40 bp	Gao et al., 2022b
					Two loci (98%)			
					Three loci (81%)			
kuΔ70 for blocking NHEJ	P_ENO1_, episomal	P_tRNA3_, episomal	100%	/	Single gene (89.7%)	No	50–500 bp	Dalvie et al., 2022

^1^HR, homologous recombination.

^2^HA, homologous arms.

### Selective marker and marker recycling

In addition to functional studies regarding gene deletion, there are many kinds of knock-out strains that have been applied in the field of industrial biotechnology (Ito et al., 2018; Karbalaei et al., 2020; Aw et al., 2021; Guo et al., 2021). However, targeted gene knock-out of *P. pastoris* is still time-consuming (Ahmad et al., 2019). Typical genomic integration as well as gene replacement by HR expression cassettes for selecting markers was used extensively caused by a large number of recent patents in the field of genome editing based on CRISPR/Cas9 (Fischer and Glieder, 2019; Liao et al., 2021). Considering the shortage of selective markers, multiple genetic modifications of *P. pastoris* are limited (Liao et al., 2021). In the past, approaches are usually combined with zeocin-resistance marker recycling vectors that employ FRT/FLP recombinase or Cre/loxp recombination system (Li C. et al., 2017), which removes the marker sequences between two recognition sites (Kobalter et al., 2022). Recently, acetamidase (amdS) was found as a kind of effective selection marker for the integrative transformation in *P. pastoris* when requiring many times of gene deletion or insertion. It was allowed to easily recycle markers by counter-selection with fluoroacetamide (Piva et al., 2018). However, after the employment of the CRISPR/Cas9 system, it is also easy to achieve such recycling by CRISPR/Cas9 geneticin plasmids that contain gRNAs for targeting the Zeocin resistance gene. *P. pastoris* strains generated by this approach can be retransformed by Zeocin resistance markers that contain plasmids (Yang et al., 2020). Marker-free system was also possible by the use of multi loci gene integration approach mediated by CRISPR/Cas9 with efficient gRNA targets in *P. pastoris*. The use of high efficient sites with a 100 bp range of upstream promoter and downstream terminator enables the integration of multiple gene cassettes into genome simultaneously (Liu et al., 2019).

### Plasmid

Plasmids can be usefully observed in bacteria, but only in a few yeast species. Generally, an episomal plasmid was not considered as an optimal choice to express the heterologous genes because of segregational instability in non-selective conditions, but it is an excellent choice regarding the expression of Cas9 (Pena et al., 2018). However, episomal plasmids for heterologous gene expression and the CRISPR/Cas9 system for genome editing have not been well developed in *P. pastoris* (Gu et al., 2019a). Based on previous attempts to apply the CRISPR/Cas9 system in *P. pastoris*, Yang et al. (2020) developed a CRISPR/Cas9 system that contained episomal sgRNA plasmid, which reached 100% genome editing efficiency in 14.7% of sgRNA. High multicopy gene editing and stable multigene editing efficiency were obtained which did not exhibit a sharp decrease due to the multi-sgRNA expression cassette. Besides HR efficiency, the vector carrying sgRNA expression cassette could be eliminated easily, benefiting the editing of other genes (Yang et al., 2020). It is necessary to identify two genetic elements, namely, the centromere and the autonomously replicating sequence (ARS), to engineer a steady episomal plasmid in a microorganism without carrying a plasmid (Piva et al., 2020; Schultz et al., 2021; Gatjen et al., 2022). Several recently identified ARS enable maintenance of episomal plasmids and efficient expression of recombinant proteins (Camattari et al., 2016; Schwarzhans et al., 2017; Nakamura et al., 2018; Gatjen et al., 2022). For instance, the episomal plasmids can be maintained in *P. pastoris* by a 452 bp DNA sequence (pan ARS) recovered from *Kluyveromyces lactis* (Camattari et al., 2016). The presence of ARS activity was found in a short fragment (111 bp) of chromosomal centromere 2 (PpARS2) (Nakamura et al., 2018). In addition, it was discovered that a piece of mitochondrial DNA (mitoARS) was able to help stabilize maintenance in the nucleosome (Schwarzhans et al., 2017). Gu et al. (2019a) thoroughly compared these ARSs, especially for creating and improving the genome editing system based on CRISPR/Cas9. Compared to a previously described method that used endogenous ARS (PARS1), CRISPR/Cas9 genome editing using panARS demonstrated an increased editing efficiency by over ten times (Weninger et al., 2016; Gu et al., 2019a). Similar findings were also discovered when establishing a *Pichia* surface display (PSD) system. Highest display levels were achieved with panARS-based expression vectors (Gatjen et al., 2022).

The engineering of microorganisms is now led by the reduction of manufacturing costs, mainly reached by higher titers. To efficiently enhance the protein production by *P. pastoris*, a fusion protein that includes the alpha mating factor secretion signal, researchers constructed human serum albumin (HSA) for promoting the protein secretion in *P. pastoris* (Sigar et al., 2017; Cao et al., 2018). The most recent recombinant proteins produced by *P. pastoris* were referred to below in Table 2.

**TABLE 2:** The most recent recombinant protein expression systems in *Pichia pastoris*.

Vector name	Used strain	Promoter	Recombinant protein	Yield	References
pPICαA-rlys	X-33	AOX1	Lysostaphin	1,141 mg/L	Shen et al., 2021
pPIC9k	GS115	AOX1	Human epidermal growth factor (hEGF)	2.27 mg/L	Eissazadeh et al., 2017
pPICZαA	GS115	AOX1	Antimicrobial peptide (Hispidalin)	98.6 mg/L	Meng et al., 2019
pPICZαA	GS115	AOX1	Antimicrobial peptide (PaDef)	unknown	De-Mei et al., 2017
pPIC9K	GS115	AOX1	Aspartic proteases	4.5 g/L	Qian et al., 2018
pAO815	SMD116	AOX	Manganese peroxidase (MnP)	126 mg/L	Xu et al., 2017
pPICZ	KM71H	AOX1	Human ATP-binding cassette	0.5 mg/L	Lee et al., 2017
pGAPZ	GS115	GAP	Tachyplesin 1 (TP1)	27.24–29.53 mg/L	Lia et al., 2019
pPICZαA	X-33	AOX	Human telomerase inhibitor 1 (hPinX1)	unknown	Unver et al., 2018
pD902	BG10	AOX1	Human papillomavirus type 52	128.015 mg/L	Dewi et al., 2022
pPICZαB	GS115	AOX1	Dengue virus NS1 protein	2.18 mg/L	Allonso et al., 2019
pPICZαA	GS115	AOX1	Recombinant α-carbonic anhydrase	47.5 mg/L	Faridi and Satyanarayana, 2018

## Promoter engineering

The widely used promoters included AOX1, GAP, FLD1, ICL1, YPT1, NPS, which are usually classified into two groups: synthetic and natural promoters (Juturu and Wu, 2018). As natural promoters showed a limited capacity to tune expression levels and regulatory characteristics, many promoter engineering initiatives have been made for *P. pastoris*, especially for P_AOX1_ (Vogl, 2022). Besides P_AOX1_, some other methanol-inducible promoters have been characterized in *P. pastoris*. However, when considering the usage of methanol, it is highly inflammable and toxic. Using methanol for the high-level protein expression in the food and pharmaceutical industry is not favorable (Juturu and Wu, 2018). Some methanol free expression systems were designed for specific applications. On the other hand, synthetic promoter is becoming a trend for the improvement of protein production.

### P_AOX1_ engineering

The promoter of the *P. pastoris* alcohol oxidase 1 gene (P_AOX1_) remained the most commonly used natural promoter for the RP expression vector construction in *P. pastoris* (Vogl and Glieder, 2013). It can be strictly inhibited by glucose or glycerol and triggered by methanol, which enables cells to grow in methanol as solely carbon source. In other words, it decouples cell growth and protein production phase. Relying on these mentioned advantages, it is of big value for the high-level protein expression and can replace the constitutive P_GAP_ (promoter region of the glyceraldehyde-3-phosphate dehydrogenase) in certain cases (Gellissen, 2000).

The clarification of regulation mechanism promoted the development of P_AOX1_ engineering. Methanol expression regulator (Mxr1) essentially regulates the utilization pathway of methanol and is capable of activating a lot of genes in response to methanol (Wang et al., 2016). The overexpression of Mxr1 has the function of promoting AOX1 expression through inhibiting glycerol transporter 1 (GT1) expression (Zhan et al., 2017). Partial rewiring of P_AOX1_ transcriptional circuits can still maintain a basic output by overproducing a deregulated Mxr1 form in strains containing multiple P_AOX1_-based expression cassettes (Camara et al., 2019). Targeted editing of Mxr1 can be successfully achieved by CRISPR/Cas9 technology with plasmids containing sgRNA and the methanol expression regulator 1 homology arms (Hou et al., 2020). In other words, Mxr1 protein frame-shift mutations may reduce AOX1 protein levels as well as weaken the enzyme activity (Hou et al., 2020). In one study, the P_AOX1_ engineering by a mutation in the core promoter where wild type adjacent triplets were changed to cytosine or adenine triplets, a completely synthetic construction, demonstrating the strong tolerance of the core promoters to small mutations, which supported regulatory models regarding degenerate motifs, or redundant design in the future (Portela et al., 2018). Generally, modifications in P_AOX1_ core promoter or around upstream regulatory sequences (URS) by synthetic promoter demonstrate regulatory effects, and the engineering effort is mostly focused on 5′ untranslated regions (5′ UTR) (Vogl and Glieder, 2013). In a more recent study, the genetic modifications of P_AOX1_ were realized by using synthetic Adr1, Cat8, and Aca2 *cis*-acting DNA elements to replace specific *cis*-regulatory DNA elements for Mxr1, Cat8, and Aca1 binding, respectively. The combined promoter design with 3 Cat8, 3 palindromic Adr1, as well as Aca2 synthetic binding motifs can retrofit the strength of methanol to 1.97-fold that of P_AOX1_ (Ergün et al., 2020).

### Alternative methanol-inducible promoters

Besides P_AOX1_, some other methanol-inducible promoters have been characterized in *P. pastoris*. The expression level of the promoter of the dihydroxyacetone synthase 2 gene (P_DAS2_) was higher than that of P_AOX1_ (Vogl and Glieder, 2013), while the expression of P_PEX8_ and P_AOX2_ was much lower (Vogl, 2022). In order to identify alternative methanol inducible promoters, the transcriptional response of *P. pastoris* on microarray was observed under various carbon source conditions including glucose repression, derepression, and methanol-induction (Vogl et al., 2016). Fifteen different strengths of methanol-responsive promoters that participate the methanol utilization (MUT) pathway were identified. Clearly, the promoter of the CAT1 gene participating in the defenses against ROS, presented a strong methanol induction and the highest derepression level. P_CAT1_ induction can be achieved by using oleic acid at a similar level to methanol. Therefore, P_CAT1_ could be considered as an appropriate alternative derepressed, methanol-free promoter if its regulation mechanism was to be further elucidated (Vogl et al., 2016). Orthologous promoters from related yeast species were tested and even surpassed endogenous promoters in *P. pastoris*. Under methanol induction, the promoter of the *HpMOX* gene from *Hansenula polymorpha* (Hp) presented a similar expression levels as P_AOX1_ and *HpFMD* promoter surpassed P_AOX1_ by threefold (Vogl et al., 2020). These results indicated the potential of utilizing high-efficacy orthologous promoters from other eukaryotic hosts. Similar findings were revealed in the terminators, the terminator sequences from *S. cerevisiae* were confirmed to maintain function when transferred to *P. pastoris* (Vogl et al., 2020).

### Methanol free expression system

While methanol induction offers strong promotion and regulation of recombinant protein expression, it also contributes its own challenges. Due to the safety concern and strict process control for inducing methanol in large-scale fermentation, there have been some efforts in terms of the replacement of methanol as a single carbon source in *P. pastoris* (Wei et al., 2016). After the deletion of three transcription repressors and overexpression of one transcription factor MIT1, one methanol-free P_AOX1_ start-up strain was successfully constructed (Wang J. J. et al., 2017). Accordingly, a glucose-glycerol-shift induction model was built for replacing conventional glycerol/methanol induction in the wild-type strain. It is safe, economic, and environmentally friendly, but exhibits less protein expression ability. Only 77% GFP expression level was detected in glycerol relative to the wide type in methanol (Wang J. J. et al., 2017). Later, another study confirmed that any P_AOX1_-based strain can be converted to a methanol-free system with the simple overexpression of Mit1 or Mxr1 by derepression of P_CAT1_, and no addition of an alternative inducer was required, however, low feasibility and stability remain to be the main concern of this method (Vogl et al., 2018b). As one transcription regulating element in the MUT gene of *P. pastoris*, P_DAS1_, a strong methanol-inducible promoter driving dihydroxyacetone synthase expression, is also considered to be applied to RP production (Vogl et al., 2021). Constitutive expression of one single transcription factor *KpTrm1* was enough to activate P_DAS1_ without the addition of methanol, the simplicity of P_DAS1_ regulation made it promising for the development of methanol-free protein expression system (Takagi et al., 2019). The protein yield remains to be a drawback in terms of these innovative methanol free system.

### Novel synthetic promoters

Despite the limitation of the selection of protein expression promoters in *P. pastoris* to P_AOX1_ or P_GAP_ (Yang and Zhang, 2018), researchers have made efforts to find new synthetic promoters that are likely to replace conventional promoters. Engineered promoter variants (EPVs) exhibit an extremely stronger performance than natural promoters and allow to conduct “green-and-clean” production on a non-toxic carbon source in the first-choice utilization pathway of carbon source of yeast. One recent work to improve the RP expression with ethanol utilization pathway was done by Ergn et al. (2019). Transcription binding sites of alcohol dehydrogenase 2 promoter (P_ADH2_) were engineered for designing the novel engineered promoter variants (NEPVs) (Ergn et al., 2019). The most popular method for the generation of EPVs is hybrid-promoter architectures. Using *de novo* synthetic sequence to replace native *cis*-acting DNA assists in achieving the architecture of the hybrid promoter (Ergün et al., 2020). The hybrid-architectured promoter design refers to collecting monodirectional double-promoter expression system (DPESs) with hybrid architecture composed of engineered promoter variants P_ADH2_-Cat_8_-L_2_ and Pm_AOX1_ and the natural promoter P_GAP_ for enhancing and upregulating deregulated gene expressions in *P. pastoris* in media free of methanol (Demir and Calik, 2020). Biofunctional DPESs exhibited higher transcription and expression upregulation power relative to twin DPESs (two-copy expression systems). The most advanced technology of *P. pastoris* promoter engineering provides the comprehensive insights into the *cis*-acting DNA motifs and functions to ensure a rationally designed non-conventional promoter libraries that possess improved strength and different regulation mechanisms (Ergun and Calik, 2021). Apparently, synthetic promoter variants are becoming a trend for improvement of protein expression in the *P. pastoris* system (Machens et al., 2017). Lastly, since suitable promoters can mediate dynamic regulation of biosynthetic pathways and maintain cellular robustness, the appearance of the inexpensive synthetic promoters still remains to be explored for future applications (Vogl et al., 2016).

## Metabolic engineering

### Engineering of protein glycosylation

Metabolic engineering regarding *P. pastoris* initially featured protein glycosylation humanization (Irani et al., 2016; Pena et al., 2018). There are two main types of glycosylation, including N- and O-glycosylation. Researches show that N-glycosylation had a great effect on protein stability (Zou et al., 2013), activity (Zou et al., 2012), and specificity (Yang et al., 2015). However, the removal of N-glycosylation didn’t make great changes on the secondary structure of proteins but tertiary structure showed differences (Wang et al., 2018). Another recent study found that the degree of O-glycosylation was remarkably higher when the protein was induced by methanol compared with glucose (Radoman et al., 2021), but details of machinery required to be investigated later.

### Metabolic flux analysis

**FIGURE 1:**
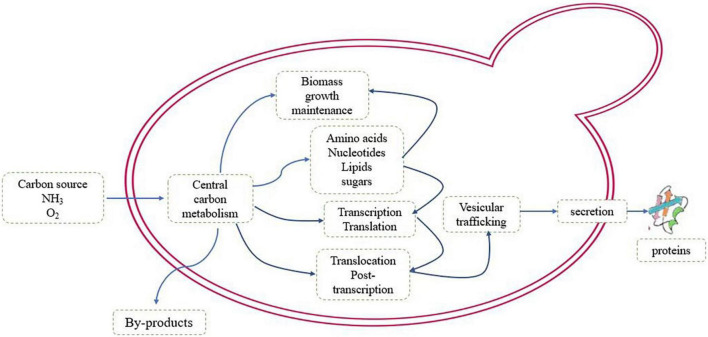
The schematic of metabolic pathway of the recombinant protein production.

In view of the proven history of *P. pastoris* as an industrial cell factory for protein production, there has been an increasing interest in exploring its potential to produce primary and secondary metabolites. The RP production is a costly metabolic process, and during the process, many cellular machineries deviate from the targets of evolution regarding cell development and maintenance, precursors substances are expelled from the central carbon metabolism, and redox and energy cofactors are consumed, leading to low energy efficiency in the metabolism (Kafri et al., 2016). Thousands of biochemical reactions occur coordinately, hence cells are capable of obtaining energy as well as building blocks from the environment for maintaining their lives (Ergun et al., 2021). The metabolic constraints of recombinant protein production are shown in Figure 1.

The accuracy of metabolic flux analysis depends on the biomass composition, which is obviously different in cells grown on various carbon sources. The differences of biomass composition were found to remarkably affect the lipid flux distribution, the biosynthesis of secondary alcohol and the oxidation-reduction processes (Cankorur-Cetinkaya et al., 2017; Tomas-Gamisans et al., 2018). Considering the abundant proteinogenic amino acids, NMR can serve for evaluating how RP production affects the metabolomic profiles (Tredwell et al., 2017). On that account, correlation between these unfolded protein response (UPR) markers and specific metabolic signals was unveiled by transcript analysis of gene transcripts associated with UPR (HAC1, KAR2, PDI1) (Tredwell et al., 2017). This approach allows for the high-throughput screening of large numbers of clones in the future.

### Metabolic engineering to enhance protein production

In spite of the achievements in recombinant DNA technology, the protein expression by engineered *P. pastoris*, faces many limitations, indicating that expression system exhibits unpredictable physiology (Prabhu et al., 2017). Above protein production restrictions can be solved *via* the metabolic engineering strategies through increasing the precursor supply as well as enhancing the cellular redox and energy efficiency. Protein folding and processing in the endoplasmic reticulum (ER) mainly restrict the protein production and secretion from *P. pastoris*. Recombinant gene overexpression is capable of destroying the inherent secretory machinery regarding *P. pastoris*, and accumulation of proteins folded in incorrect way occur inside the ER. For restoring the normal protein folding, an UPR pathway was triggered naturally by cells, causing expression upregulation on genes coding for chaperones as well as other folding-assisting proteins (Raschmanova et al., 2021). From another application perspective of metabolomics, metabolic biomarkers could indicate the cellular stress resulted from the UPR under when the expression was high (Tredwell et al., 2017). The effect of vacuolar protein sorting (VPS) system on protein secretion was investigated by generating VPS mutant strains. The impairment of VRS is considered as an effective means of enhancing recombinant protein secretion (Marsalek et al., 2017). The N-terminal portion of the pre-pro-α-factor is a common secretion signal, but it is capable of partially leading to aggregation, hence limiting the export from the ER (Barrero et al., 2018). A hybrid secretion signal that possessed the *S. cerevisiae* Ost1 signal sequence was engineered in *P. Pastoris* to facilitate co-translational translocation into the ER, and then the α-factor pro region (αPR). When Ost1 signal sequence was paired with the αPR, its secretion efficiency was much higher relative to the α-factor signal sequence (Barrero et al., 2018).

### Genomic integration

The manipulation of genes gives more possibilities for metabolic engineering through different carbon utilization pathways. As described before, by overexpressing only one transcription factor such as MIT1 in P_AOX1_-based expression strains, methanol-dependent strains could be transferred into a glucose/glycerol-regulated system (Vogl et al., 2018b). Furthermore, after adding 8 heterologous genes as well as deleting 3 native genes, it is possible to observe the transformation of the traditional *P. pastoris* methanol-assimilation pathway into a CO_2_-fixation pathway similar to the Calvin–Benson–Bassham cycle (Gassler et al., 2020), which provides us more insights in terms of innovating novel green and carbon-neutral process. Most recently, in order to regulate the target gene expression level of metabolic pathway, fast and reliable tools are applied to precisely manipulate the expression of target genes. One practical example was dCas9, which can be fused *via* RNA scaffolds to *trans*-activator domains and thus regulate the gene expression when targeted to the promoter region of a gene (Baumschabl et al., 2020).

## Mathematic models

The mathematic modeling regarding *P. pastoris* used for protein production mainly pays attention to 2 overall objectives: (1) predict nutrient and oxygen-related cell growth mainly for the design and control strategy of bioprocess; (2) enhance the understanding of metabolic responses under operational and nutritional conditions, thereby improving the productivity. The cell behavior was modeled to be a sub-system in a bioreactor, which may make the modeled system boundary confined to the bioreactor, cells, or both. Despite the different methods and mathematical tools used for fulfilling above objectives, a majority of them take into account the solutions of the linear equation (LE) systems or the ordinary differential equation (ODE) systems (Theron et al., 2018).

The earliest metabolic network of *P. pastoris* model came up with central carbon metabolism, using this model to calculate the ^13^C metabolic flux distributions (Maaheimo et al., 2001). The metabolic insights based on information of intracellular flux balances allow to build the metabolic model to be a useful tool before confirming the bioreactor process, for conducting partial prediction on the impact exerted by certain genetic modification (expression deletion or over expression) related parameters on the strain behavior (Kerkhoven et al., 2015; Cankorur-Cetinkaya et al., 2017). Recent years, major breakthroughs have been made in the systematic quantitative analysis of its physiology. Genome-scale metabolic models (GSMM) can be developed relying on the abundant information, hence, new approaches can serve for the engineering of host cells and bioprocess (Barrero et al., 2018). Incorporating heterologous protein production into a GSMM allows to simulate the influence of the overproduction as well as predict the improved productivity relying on the deletion or the overexpression of gene targets. Studies reported the ability of GSMM to predict the overexpression and deletion mutants that can improve the production of RP with high accuracy (Nocon et al., 2014). Taking advantages of available genomic information, thousands of metabolites and reactions are extended into the stoichiometric matrix, and GSMMs are configured (Famili and Palsson, 2003). Overall, the stoichiometric matrix imposed above constraints, and one or more flux values usually exhibited an association with the steady-state carbon source consumption rate of carbon source (Yasemi and Jolicoeur, 2021). With the help of GSMMs, the cell growth can be predicted with the supply of different nitrogen and carbon sources, the distribution of metabolic flux, as well as the way gene deletion or overexpression affects cell growth. Even the metabolic effects of some culture parameters on microbial cell factory performance like dilution rates and oxygen levels can be evaluated with the elucidation of their metabolic consequences (Torres et al., 2019).

Unlike the changes caused by biomass composition in the metabolic network, it was observed in a consensus genome-scale model that the variances of biomass composition didn’t make great differences to gene essentiality (Cankorur-Cetinkaya et al., 2017). The accuracy of mathematic models depends on the identifications of different reactions, meanwhile emphasizing the rankings of the altered candidate targets. The consistency and standardization of models decide whether they are appropriate for being reused (Cankorur-Cetinkaya et al., 2017). After the use of iterations, the dynamic block of the aforementioned states can be identified. The substrate uptake kinetics were determined by a kinetic block and the flux distributions were determined by a metabolic block (Sanchez et al., 2014). However, genome-scale modeling has the disadvantage of lots of simulations which was very complex in terms of the iterative method (Saitua et al., 2017). Oppositely, algorithmic optimization could serve for optimizing the protein productivity relying on the Dynamic flux balance analysis model (dFBA) (Sanchez et al., 2014). This model includes bilevel optimization, penalization schemes, and direct dynamic optimization, supplying the dynamic simulation of the extracellular bioreactor environment and intracellular fluxes in *P. pastoris* at the same time, and thus provide practical simplification and optimization (Emenike et al., 2018). The study investigated the RP erythropoietin, finding that the use of a small metabolic network avoided the iterative approach of GSMM (only 47 intracellular reactions relative to 1,361 reactions in the GSMM) (Emenike et al., 2018). Besides, considering the certain bioprocess restrictions as well as the strain features, the detailed relationship of specific growth rate with volumetric productivity can be predicted ahead of the practical process (Saitua et al., 2017). A larger amount of annotated genes, reactions, metabolites, as well as reaction locations can help improve the metabolic coverage and prediction accuracy of a *P. pastoris* GSMM, and assist in carefully adding the biosynthesis pathways regarding cofactors and vitamins for a better improvement (Ye et al., 2017).

In terms of bioreactor modeling, the elements required for construction include matrix, biomass, total protein, other medium components, and off-gas constituents. The probability distribution predicted by the model quantifying parameter distributions can be quantifiably consistent with the inter-run variability observed in the experimental data. Unlike GSMM, model prediction requires experimental data under different operating conditions. The improvement of chemical defined medium can be realized by the minimization of the number of components required while meeting cellular requirements (Hong et al., 2021).

## High throughput screening

As we still lack much knowledge about inner reactions and cellular responses. The accuracy of mathematic modeling cannot be ensured. The low efficiency of various modifications requires for rapid and functional perturbations. High throughput screening is an advanced and rapid laboratory technique that has been used in many microorganisms before (Sjostrom et al., 2014; Kenzom et al., 2015; Clare et al., 2019). The identification of desired results combined with the appliance of reverse engineering can enable the attainment of strains carrying specific mutations associated with the desired phenotype (Wang G. K. et al., 2017). High-throughput screening method can be simplified by carrying out the growth phase and induction phase together by inoculation of *P. pastoris* clones directly into methanol-containing medium (Kenzom et al., 2015). In addition, high-throughput screening help understand the impact of genetic engineering such as ectopic integration. It is possible to neglect the impact exerted by the integration locus in the promise of comparing enough transformed strain number (Vogl et al., 2018a). With the generation of 168 synthetic bidirectional promoters (BDPs) in *P. pastoris* and the leverage of naturally occurring BDPs as a parts repository, different expression profiles and ratios were fast screened for optimizing the gene co-expression. This library strategy depends on the highly conserved BDP architecture of histones and can be extended to other eukaryotes (Vogl et al., 2021).

Flow cytometry is considered as a powerful, high throughout technology that provides rapid muti-parametric analysis of single cells in solution (McKinnon, 2018). It was primarily used for measuring the fluorescence intensity produced by fluorescent-labeled antibodies which served for detecting proteins or ligands that bind to various molecules related to cells. Specially, it was used for quantifying the physiological state regarding *P. pastoris* in heterologous protein production process in cultures with high cell density (Zepeda et al., 2018). Thousands of individual cells were measured simultaneously by the use of fluorescent probes and several parameters (Cossarizza et al., 2019). By using flow cytometry, not only were sedimentation and possible agglomerations of biased cells minimized, but a false-positive detection of loosely agglomerated cells could also be minimized due to the in-flow velocity of the cell suspension and the force exerted on the cells (Pekarsky et al., 2018). As an at-line, single-cell and non-invasive method, flow cytometry could be applied for evaluating the unfolded protein response (UPR) and cell viability. The observed upregulation of UPR didn’t show any positive correlation with impaired viability (Raschmanova et al., 2019). Nowadays, with the development of deep convolutional neural network, a system for image-guided cell sorting and classification with the high throughput of flow cytometer was able to be established. The characteristics could be automatically extracted after being processed directly on the one-dimensional time-series waveforms (Gu et al., 2019b).

The establishment of high cell density fed-batch biomanufacturing faces difficulties in the screening phase and the earlier bioprocess development phase (mainly using shake flasks and microplates) affected by the costly and time-consuming process together with the weak experimental complexity (Totaro et al., 2021). Fortunately, microfluidics with high throughput, strong reproducibility, big parallelization, high operability, and low cost, serve as a powerful platform for analyzing SCPs (Chen et al., 2019). The high-throughput (100,000 strains/hour) *P. pastoris* screening system that combines the single-cell droplet microfluidic control is capable of screening a library of million strains within 10 h by consuming a small amount of (100 μL) fluorescent reagent. Compared with traditional microplate screening, the reagent cost was reduced by millions of times (Lu et al., 2019). With atmospheric and room temperature plasma (ARTP) mutagenesis and droplet-based microfluidic high-throughput screening, it is possible to explore the potential of protein production by employing iterative rounds of genome-wide diversity generation (Kenzom et al., 2015; Ma et al., 2019; Yuan et al., 2022). For example, compared to the beginning strain, the best mutant strain showed a twofold increase in cellulase activity in one research (Yuan et al., 2022). One high-throughput platform was established, which could process any protein with a His6-tag. To selectively preserve any protein of interest, vast genetic libraries of strains were encased in biocompatible gel beads using droplet microfluidics. Then, flow cytometry discovered strains with higher production titers after the fluorescent labeling of bead-retained products (Napiorkowska et al., 2021). Overall, the application of high throughput screening enabled massively parallel characterization of microorganisms and accelerated the costly and time-consuming process of strain engineering.

## Conclusion

The development of some modern and powerful genome editing techniques engineered the expression system in a very efficient way and highly improved the HR efficiency. It is allowed to select a proper strain that accords with expectations from modeling or one that serves as a high-promise candidate relative to others. Breakthrough of these technologies makes the characterization of host strains much easier than before. The integration of promoter engineering promoted the expression level to be more controllable. Due to the safety concern raised by methanol, some novel promoters have been created for developing a methanol-independent expression system. Theoretically, it is allowed to improve the RP production in *P. pastoris* in methanol-free media through (a) the activation of existing pathway (s), or the generation of new regulatory circuits relying on engineered AOX1/2 promoters, or (b) the design of synthetic new pathways with different transcription factors.

More deeply understanding the metabolic responses can assist in directing the attempts for strain customization *via* the genetic engineering for pathway modifications. Before constructing the production process, the investigation of the metabolic engineering of the organism can help to understand the response of cells to the envisioned process. In particular, a genome-scale model can manage all the required information for the improvement of protein secretion in the design of cell factories. As the complete genome sequences are available and systems biology makes advances, genetic manipulation more frequently serves for modifying the certain cellular biochemical reactions to enhance the protein production. Despite this, the newest experimental data and more annotations are needed for a continuous upgrade and validation on the predictive accuracy exhibited by the well-recognized model previously. With the help of mathematical modeling, it would be easier to have a comprehensive understanding of the whole process and optimize key factors which influence the final yield and target productivity of proteins. There is no doubt that *P. pastoris* has better performance over *Escherichia coli*, *S. cerevisiae*, baculovirus/insect cells, SFV or adenovirus/mammalian host cells specific to soluble, secretable protein production. Relative to other prokaryotic and eukaryotic expression system, it boosts the advantages of (Prielhofer et al., 2013; Gupta and Shukla, 2017):

• Grow easily and fast to a high cell density in the defined medium;
• High level of productivity in an almost protein free medium;
• Easy to conduct genetic manipulation of yeast expression vectors with good characteristics;
• Easy of diverse post-translational modifications;
• Allow to purify secreted proteins from growth medium with no need to collect the yeast cells themselves;
• Free of endotoxins and virus.

Although *P. pastoris* was thought to be a good platform strain for RP expression, this type of system features proteolytic degradation and product truncation, which results in lower yields and less biological activity (Sinha et al., 2005). Fundamental knowledge of *P. pastoris* is relatively limited compared to that for *S. cerevisiae*, and there are fewer available molecular tools used for *P. pastoris* like terminators, promoters, or knockout strains (Dikicioglu et al., 2014). The expression level for most proteins still remains to be improved for the requirement of industrial use. Although RPs from yeast are likely to be less useful in biopharmaceutical applications due to the emergence of hyper-mannosylation, it was reported that the glycoengineered *P. pastoris* strains could produce RPs with highly uniformed human N-linked glycans (Potgieter et al., 2009). However, It is expected that the capacity of protein production by *P. pastoris* can be highly improved later and show more advantages over other yeasts.

## Author contributions

YP: data collection, investigation, writing – original draft, and review and editing. JY: review and editing (supporting) and proofreading. JW and LY: supervision. HF: main supervision, revising, reviewing, and proofreading. All authors contributed to the article and approved the submitted version.
